# A flow through device for simultaneous dielectrophoretic cell trapping and AC electroporation

**DOI:** 10.1038/s41598-019-48198-x

**Published:** 2019-08-19

**Authors:** Meera Punjiya, Hojatollah Rezaei Nejad, Juanita Mathews, Michael Levin, Sameer Sonkusale

**Affiliations:** 1 Tufts University, Department of Electrical and Computer Engineering, 161 College Ave, Medford, MA 02155 USA; 2 Nano Lab, Advanced Technology Laboratory, 200 Boston Ave, Medford, MA 02155 USA; 3 Allen Discovery Center at Tufts University, Department of Biology, Medford, MA 02155 USA

**Keywords:** Biotechnology, Engineering

## Abstract

Isolation of cells and their transfection in a controlled manner is an integral step in cell biotechnology. Electric field approaches such as dielectrophoresis (DEP) offers a more viable method for targeted immobilization of cells without any labels. For transfection of cells to incorporate exogenous materials, electrical methods such as electroporation, are preferred over chemical and viral delivery methods since they minimally affect cell viability and can target many types. However prior approaches to both methods required multiple excitation sources, an AC source for DEP-based trapping and another DC source for electroporation. In this paper, we present a first of its kind flow through lab-on-chip platform using a single AC excitation source for combined trapping using negative dielectrophoresis (nDEP) and AC electroporation. Use of AC fields for electroporation eliminates the unwanted side effects of electrolysis or joule heating at electrodes compared to DC electroporation. Adjusting the flow rate and the electrical parameters of the incident AC field precisely controls the operation (trap, trap with electroporation and release). The platform has been validated through trapping and simultaneous transfection of HEK-293 embryonic kidney cells with a plasmid vector containing a fluorescent protein tag. Numerical scaling analysis is provided that indicates promise for individual cell trapping and electroporation using low voltage AC fields.

## Introduction

Analysis of cells at the single-cell level has become increasingly important for drug discovery, cancer genomics and discovery of new pathways^1^. Platforms developed for such single-cell analyses require a mechanism for cell separation, confinement and controllable transfection of those cells. Several label-free methods have been demonstrated at the microscale for cell separation including deterministic lateral displacement^2^, deformability-based separations^3,4^, inertial-based separations^5^, acoustophoresis^6^, optical tweezers^7^ and dielectroporesis (DEP)^8,9^. While all viable for sorting, only the latter two have been demonstrated as a viable methods for both cell separation and confinement.

Optical tweezers use a high power laser to exert forces on dielectric particles determined by the relative refractive index of the particle and the surrounding medium. Typically, a high power laser is focused using a microscope objective inducing an electric field gradient at the beam waist. This forces dielectric particles up the induced gradient to regions of high electric field^10^. While demonstrated for separation and confinement, the necessary high power lasers and expensive optical setups limit the scalability of the technique.

DEP is arguably the simplest available method for both cell separation and confinement in microfluidic devices, requiring only generation of an electric field. This phenomenon uses inhomogeneous electric fields to exert a translational force on dielectric particles, similar to the Lorentz force which acts on charged particles in homogenous fields. Numerous lab-on-chip (LOC) devices have demonstrated DEP as a viable method for cell capture and immobilization^8,11–16^. For this purpose, negative DEP (nDEP) in particular has been shown to dramatically improve cell viability compared to techniques using positive DEP (pDEP)^17,18^. This is because in negative DEP, cells are manipulated to regions of low field strength, preventing large disruptions of the cells’ transmembrane potential, which can cause unwanted lysis due to irreversible poration of the cell membrane. Traditionally, the time-averaged DEP force acting on a spherical particle when placed in a polarized medium is given by Equation 1 ^19^ where *ε*_*o*_ is the permittivity of free space, *ε*_*m*_ is the relative permittivity of the polarizable medium, R is the radius of the particle, *E*_*RMS*_ is the root mean square of the AC sinusoidal electric field and CM is the Clausius-Mossotti factor given by Equation 2 and describes the relative permittivity of the particles and surrounding medium. Here, ${\varepsilon }_{ceff}^{\ast }$ is the effective complex permittivity of the cell and ${\varepsilon }_{m}^{\ast }$ is the complex permittivity of the medium. Because the DEP force is proportional to *E*_*RMS*_, both AC and DC fields can be used for DEP. In particular, when an AC field is used, the direction of particle motion is independent of the field strength and is only a function of the CM factor. When this factor is positive, the cell will experience pDEP; when it is negative the cell will experience nDEP.

1 $${F}_{DEP}=2\pi {\varepsilon }_{o}{\varepsilon }_{m}{R}^{3}Re[CM]\nabla |{E}_{RMS}{|}^{2}$$

2 $$CM=\frac{{\varepsilon }_{ceff}^{\ast }-{\varepsilon }_{m}^{\ast }}{{\varepsilon }_{ceff}^{\ast }+2{\varepsilon }_{m}^{\ast }}$$

Transfection, the latter of the required functions, can easily be accomplished through a temporary and reversible poration of cell membrane known as electroporation, which occurs under exposure of the cell to a high electric field. Here the absolute magnitude of the electric field seen by the cells will be directly proportional to the rate and size of pore formation. Once pore formation begins, the process will continue regardless of the external electric field, reaching a critical number and ceasing once the transmembrane potential reaches its steady state value, usually in less than a microseconds time^20^. Because of this, low frequency sinusoidal fields can also be used to induce electroporation, known as AC electroporation. Here, the frequency and magnitude of the electric field required to induce poration is determined by the membrane capacitance and conductivities of the medium and cell^21^.

AC electroporation has several advantages over traditional bulk electroporation including mitigation of pH changes which can occur at the electrode interface and permeation of dielectric layers required to protect non-inert electrodes from electrochemically induced corrosion^22,23^. pH changes occur at the electrode-solution interface in DC electroporation due to electrolysis which occurs at the anode and cathode. Reduction of water at the cathode causes production of hydroxyl ions creating a localized alkaline environment around the electrode^22,24,25^. In AC electroporation, electrodes alternate as anode and cathode balancing the production of both hydroxyl and hydrogen ions alleviating any pH changes which may occur at the electrodes. Additionally, in traditional DC electroporation, electrochemically induced corrosion can occur when non-ideal electrode materials such as aluminum, chromium or titanium are used. Here the metal surfaces are electrochemically oxidized or reduced to form oxides and sulfides on the electrode surfaces’. To prevent this from happening a dielectric layer can be employed on top of the electrode surface, however this prevents penetration of the DC signal and instead will expose the cells to a exponentially decaying field. AC electroporation can instead penetrate the dielectric coating without altering the applied field. This relationship is described by the Schwan Equation (3) which relates the maximal change in transmembrane potential (Δ*ψ*_*mem*_) to the frequency of the applied field^26^. Here *ω* is the angular frequency of the applied sinusoidal field and *τ* is the membrane relaxation time given by Equation 4. *R*_*c*_ is the cell radius, *C*_*mem*_ is the membrane capacitance, *σ*_*c*_ is the cell conductivity, and *σ*_*m*_ is the external medium conductivity.

3 $${\rm{\Delta }}{\psi }_{mem}=\frac{1.5{R}_{c}{E}_{amp}}{{[1+{(\omega \tau )}^{2}]}^{\frac{1}{2}}}$$

4 $$\tau ={R}_{c}{C}_{mem}(\frac{1}{{\sigma }_{c}}+\frac{1}{2{\sigma }_{m}})$$

As previously noted^27^, towards development of controlled single-cell electroporation platforms, several recent works have used DEP to monitor and manipulate cells pre- and post- electroporation, but use two distinct signal sources and sets of electrodes to complete the function, which makes scaling difficult and requires several lithographic procedures^28,29^. For example, Guo et. al. recently presented a platform for selective electroporation using nDEP positioning where four outer electrodes were used to position a cell using nDEP and an inner set of electrodes were used to electroporate cells^29^. In all, fabrication required 5 layers of aligned patterning. More recently, Sherif *et al*. proposed a platform using DEP to characterize cells before an electric field is applied, again two distinct sets of electrodes are chosen, four to position and an additional for monitoring^30^. Other works have demonstrated DEP for cell positioning and electroporation using the same set of electrodes, but required two distinct signals, one for DEP and one for EP, which cannot be used in flow-through systems if a physical means of confinement is not present^31,32^.

More recently, Lyu *et al*. completed numerical simulations for a potential device suggesting that in microscale electroporation the applied electric fields could induce AC electroporation and DEP simultaneously^27^, which could help to improve efficiency by forcing cells which would ordinarily not experience sufficient electroporation fields, to the electrode surface while simultaneously electroporating them. The proposed platform simulated interdigitated electrodes and is not suitable for single-cell trap and release. Here we present and experimentally validate a platform for simultaneous nDEP trapping and AC electroporation scalable for single-cells. Simultaneous nDEP and AC electroporation is achieved using only two electrodes with a single excitation signal. In addition to simplifying device fabrication this allows for trapping and electroporation under a continuous fluid flow, a distinct capability from previous platforms. Furthermore, inducing this co-phenomena in a controlled manner has several potential advantages in fabrication and operation.

First, in microfluidic electroporation platforms, proximity of the exogenous material to the cell becomes important as cells are only porated for a short period of time, on the order of microseconds. During this time, the material of interest will passively diffuse through the cell membrane *only* if it resides close to the cell. In our platform, cells are held fixed while porated as the solution containing this material continually flows along all sides of the porated cell, allowing for greater chance the cell will come in contact with the material of interest^33^.

Second, microscale electroporation using coplanar electrodes typically requires a physical means of confining cells to the proximity of the planar electrodes so cells will experience the necessary electric fields for poration^31^. Here, the electroporation field itself forces cells to the electrode proximity, removing the need for physical confinement. Additionally in this scheme cells are pushed to regions of low electric field with the competing drag force on the particle bringing the cells to the electroporation location, reducing the possibility of unwanted lysis. Third, in scaling to a high-throughput system with individual control, the number of electrical connections required is cut in a half, simplifying system complexity and increasing the number of sites.

In this manuscript, we present the design of a microfluidic device with an electrode geometry scalable for simultaneous nDEP and AC electroporation of single-cells. Results are demonstrated with a scaled-up version of the platform where forces exerted on the cell still hold. We validate the nDEP manipulation forces on our platform using COMSOL simulations and physical experiments with polystyrene particles. Simultaneous nDEP trapping and electroporation is demonstrated using HEK-293 human embryonic kidney cells as a model. As markers of electroporation we both observe the leaching of calcein dye from the cells and transfect cells with a plasmid for FusionRed red fluorescent protein (RFP) expression.

## Results and Discussion

### Device design and operation

**Figure 1:**
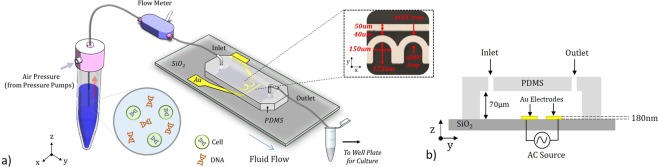
Device Overview and Setup. (**a**) The platform, consisting of gold electrodes and a PDMS microfluidic channel, is fabricated using a simple single-step lithography process on soda lime glass. Inset is an optical image of the as fabricated gold electrodes indicating dimensions and trap locations. (**b**) A cross-sectional image of device indicating channel height and electrode thickness. A single AC signal is applied between the two electrode sets in the array.

The platform, shown in Figure 1, utilizes a unique two-electrode geometry consisting of a half-ring trap with a tangential ground line. Use of this half-ring structure allows for continuous application of the electric field to trap cells under constant flow while simultaneously creating a nDEP force acting in the negative z direction, bringing cells to the trap location independent of gravity. In a fully symmetrical structure, such as a circle or square, a lifting force would have been present along the edge just inside the electrodes. Thus in a flow through system, a DEP force could have only been applied once a cell resides inside the electrodes^34,35^. The use of a linear ground electrode as opposed to concentric circles creates the largest E-field gradient along the y-z plane or along the direction of flow, resulting in an nDEP trap located inside the ring. The gold electrodes, shown in the inset image of Figure 1, have 75 *μ*m inner radius half-rings separated by 40 *μ*m from the ground lines and are fabricated using standard lithographic procedures. A porous polyethylene glycol (PEG) layer is used over the electrodes as an anti-adhesion layer. Polydimethylsiloxane (PDMS) is used to create the fluidic channel on the device which is 1 mm wide and 70 *μ*m tall. The channel is designed to be 2.34 cm in length to help stabilize pressure-driven flow within the device.

**Figure 2:**
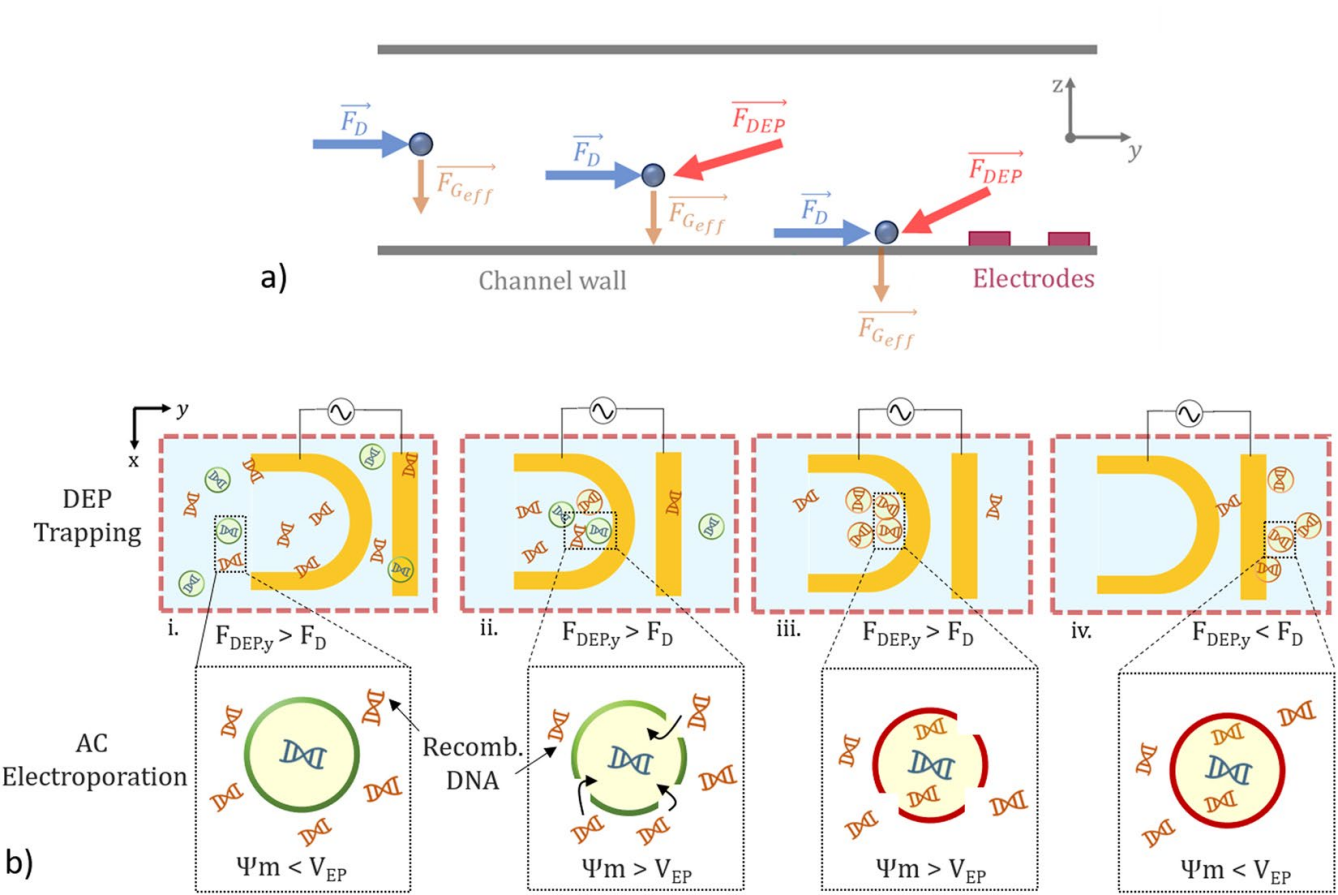
Device operational overview. (**a**) Free body diagram indicating forces present on cells within the device at various points along channel. (**b**) Operation sequence where (i) cells and material to be transfected are introduced into the system under a constant flowrate such that the y-component of the DEP force (*F*_*DEP*.*y*_) is greater than the drag force (*F*_*D*_) within the trap. (ii) As cells enter the trap they experience a dielectrophoretic force immobilizing them at a given point within the trap where the applied electric field simultaneously induces poration by causing the transmembrane potential *ψ*_*m*_ to exceed the electroporation threshold, *V*_*EP*_. (iii,iv) after cells are transfected, the flow rate is increased such that the drag force exceeds the y component of the DEP force and cells and be collected for culture.

Figure 2a indicates the forces present on particles in relation to the DEP trap. In the channel, sufficiently far from the DEP trap, the particle experiences a hydrodynamic drag force, *F*_*D*_, which will carry the particle towards the trap in the y direction. The z dimension location of the particle is determined the effective force of gravity on the particle, ${F}_{{G}_{eff}}$ which accounts for both the particle buoyancy and gravity. In operation a mixture containing the cells of interest and material to be transfected are introduced under a constant flow rate such that *F*_*D*_ is less than the DEP force, *F*_*DEP*_, while the chosen field is applied. As cells approach the nDEP trap, *F*_*DEP*_, begins to act on the cells. At some point within the trap the y-component of the DEP force, *F*_*DEP*.*y*_ and *F*_*D*_ will cancel, immobilizing the cell at the nDEP trap location. Concomitantly, as the cells enter the trap, they will begin to porate, allowing exogenous material into the cells (Fig. 2b-ii,iii). The length of time for which the field is held will determine the number of cells which are trapped and degree to which they are electroporated. To release the cells, with the field still on, the flow rate is increased such that the drag force on the cells exceeds the DEP force in the trap and cells are collected for culture and analysis (Fig. 2b-iv). For applications where simple trapping is desired, the amplitude and/or frequency of the chosen electric field can be altered.

### Finite element analysis and validation with particles

**Figure 3:**
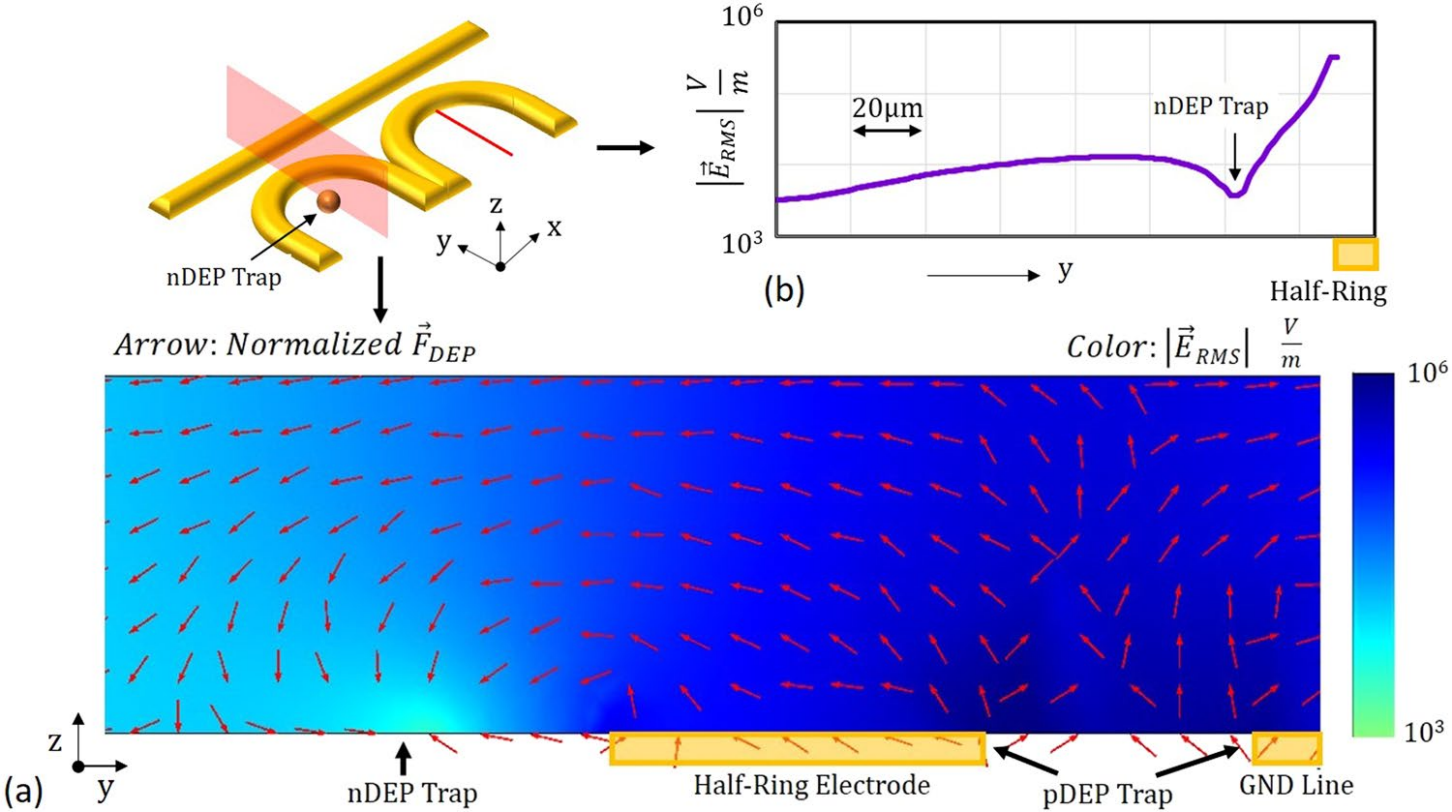
FEA Simulation and Design Verification. (**a**) COMSOL Multiphysics simulation indicating direction of the nDEP force along trap cross section indicated by the red plane. (**b**) Electric field along half-ring center line indicated by the red line.

Pure electrical simulation with a 20VPP, 1 MHz applied voltage signal between the electrodes details the location of the nDEP trap. Under no flow, the trap is located 45 *μ*m from the half-ring center along the trap’s symmetric axis as shown in Figure 3a with |*E*_*RMS*_| at the trapping point equal to approximately 4.62 kV/m (Fig. 3b). The pDEP trap is located between the half-ring electrode and ground line with the highest electric field located at the electrode edges with |*E*_*RMS*_| exceeding 259.8 kV/m. From the vector field in Figure 3a indicating the direction of the nDEP force, its clear that the trap is gravity independent as there is a nDEP force acting on the particle in the negative z direction which extends to the top of the channel. Anomalous DEP (aDEP) regions which have previously been reported in the literature were neglected as the nDEP trap location is sufficiently far from the electrode edge^36,37^.

**Figure 4:**
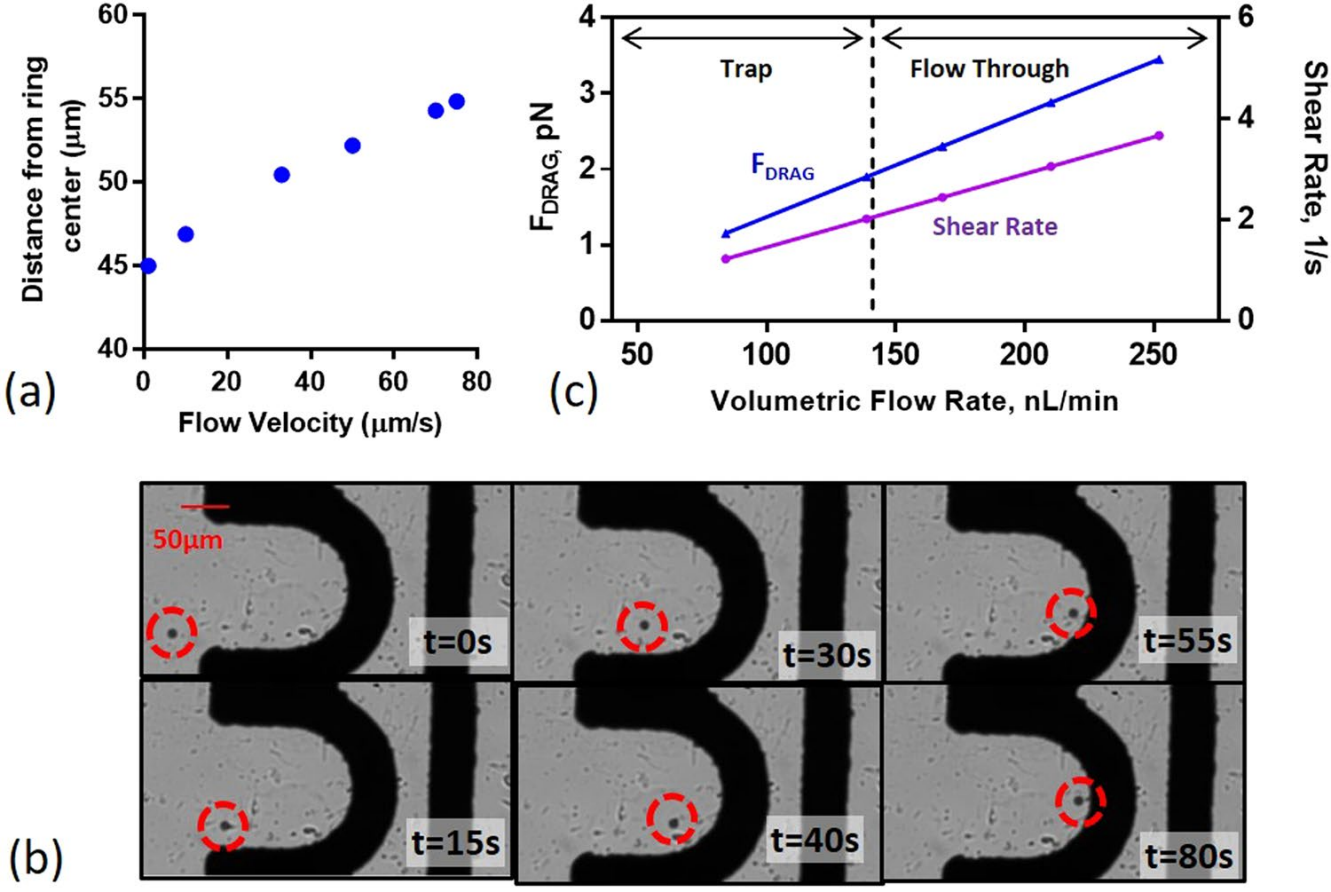
Experimental Validation with Polystyrene Particles. (**a**) Particle distance from half-ring center vs. flow velocity. (**b**) Time-lapsed images showing experimental verification of simulated particle trajectory and trap location for 9.9 *μm* polystyrene particle. (**c**) Drag force on particle vs. volumetric flow rate. Particle is held against a 140 nL/min flow rate corresponding to a 1.91 pN DEP force on the particle within the trap.

Multiphysics simulation introducing a 9.9 *μ*m polystyrene particle confirms the nDEP trap location and particle trajectory. The particle experiences nDEP subject to all field frequencies; the real portion of the CM factor vs. frequency is provided in Supplementary Figure S1. Under constant flow, the location of particle immobilization is dependent on flow velocity. Figure 4a plots the point of immobilization against the flow velocity for a 20V_PP_, 1 MHz applied signal. Supplementary Video 1 details the particle trajectory and nDEP trap location for a 9.9 *μ*m polystyrene particle held against a 33 *μm*/*s* linear flow velocity under the same electrical conditions. The particle is held where *F*_*D*_ is equal to *F*_*DEP*_ on the particle, which are both simulated to be 1.71 pN at the point of trapping. For simulation the particle is assumed to be homogeneous with uniform permittivity and conductivity throughout. Parameters used for simulation are provided in Supplementary Table S1.

The simulated DEP force within the trap is experimentally verified using the procedure outlined in Section. Time lapsed images of a particle entering the nDEP trap under a 140 nL/min flow rate is shown in Fig. 4b. Particles were held against a $\approx 141\pm 16.4\frac{nL}{min}$ volumetric flow rate for the 5 particle sample size, equivalent to a 33 *μm*/*s* linear flow velocity. Assuming simple poiseuille flow, at this flow velocity, *F*_*D*_ was calculated to be 1.91 pN and equal to the y-component of the DEP force (Fig. 4c). While the measured magnitude of the nDEP force deviates from the simulated, this can be attributed to 5% error in flow rate measurement, tolerances in channel size and tolerances in particle size which are modeled ideally in simulation. Supplementary Videos 1 and 2 provide the experimentally verified particle trajectory and the simulated particle trajectory under the same electrical and flow conditions for comparison.

### HEK-293 cells

HEK-293 cells derived from human embryonic kidney cells are used as a model for simultaneous nDEP-AC electroporation. These cells are commonly used as eukaryotic cell models for both DEP and electroporation due to their well characterized electrical and mechanical properties^38–40^. Of note, HEK-293 cells have been demonstrated with DEP for the study of transient receptor potential channels^41^, electrofusion^16^, and cell-patterning^42^.

**Figure 5:**
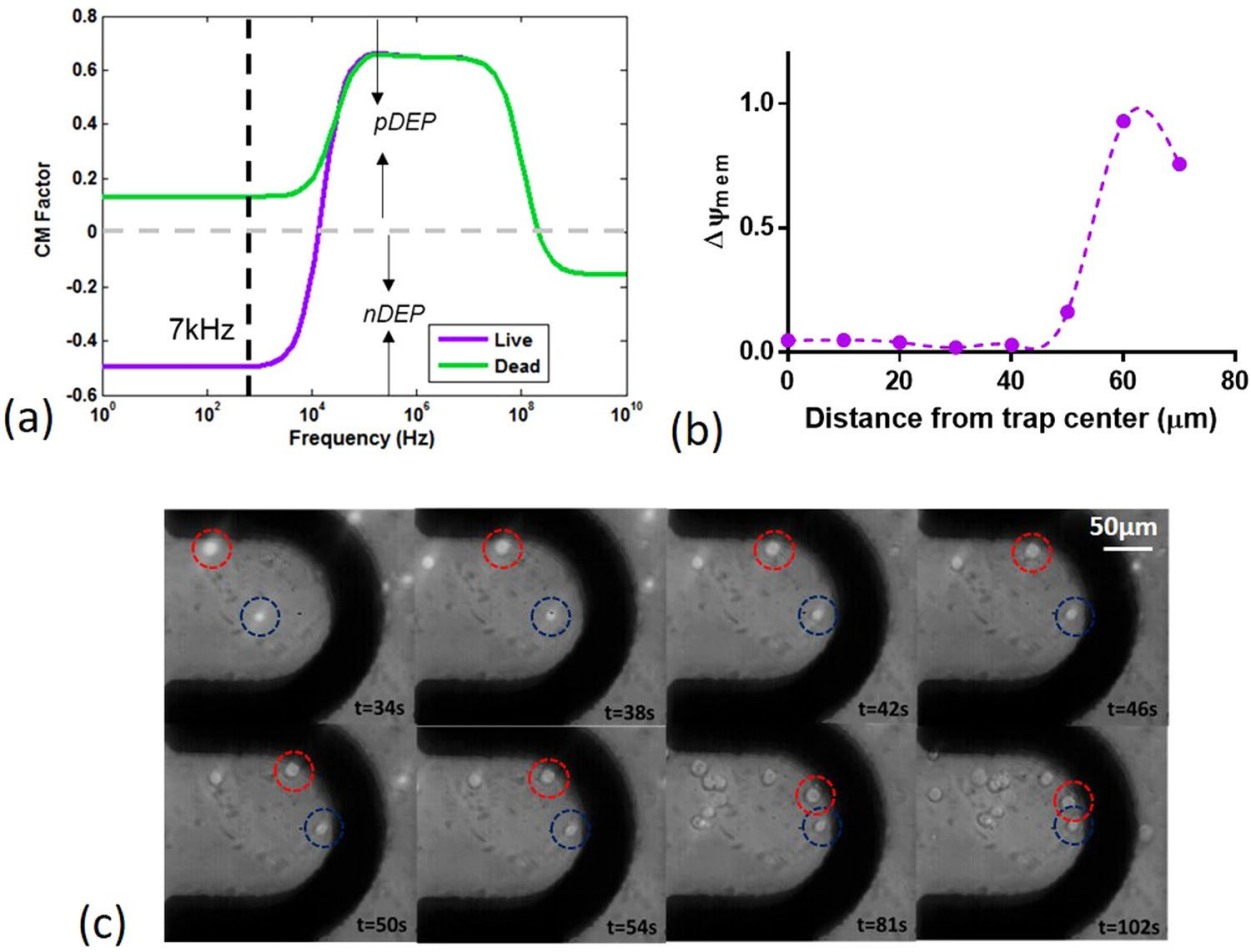
(**a**) Clausius-Mossotti factor for live and dead cells indicating live cells experience nDEP at the chosen 7 kHz frequency while dead cells will experience pDEP. (**b**) Expected change in transmembrane potential vs. distance from half-ring center for HEK-293 cells. (**c**) Time lapsed images showing nDEP trapping of HEK-293 cells. For clarity, the location of two HEK-293 cells are indicated in each time-lapsed image by the red and blue dotted circles.

For simulation, cells are modeled using the double-shell model shown in Supplementary Figure S2^19^. Parameters used for simulation are pulled from various references and are provided in Supplementary Table S2 with a detailed description of the equations used for determination of the effective complex permittivities based on these parameters. A frequency of 7 kHz is chosen to trap cells as there is a clear separation between dead and live cells at this frequency. Figure 5a shows the CM factor for dead and live HEK cells indicating live cells will experience nDEP with dead cells experiencing pDEP at 7 kHz. As the pDEP force exerted on dead cells is larger than the nDEP force exerted on live cells, this potentially allows for dead cells to remain trapped within the system as healthy cells are released.

Additionally, 7 kHz is chosen as it sits at the upper limit for AC electroporation^21^. A 4V_PP_ amplitude signal is chosen as a tradeoff such that at the point of trapping the change in transmembrane potential will exceed the electroporation threshold, assumed to be approximately 0.4 V^43^ but will not ever exceed 1.2 V at any point in the nDEP trapping region preventing unwanted lysis (Fig. 5b). Outside the regions where cells will be trapped the change in transmembrane potential is negligible such that cells will not experience poration. Thus, through adjustment of the flowrate and applied electric field, the trap can be programmed to either trap or simultaneously trap and electroporation cells.

The chosen model is coarsely verified through subjection of the cells to fields at frequencies within the negative and positive CM regions. For comparison, an image of the HEK cells subject to a pDEP force at 1 MHz is provided in Supplementary Figure S3. A time lapsed image of cells subject to 7 kHz is provided in Figure 5c. Here the location of two different cells with each time-lapse are indicated the red and blue dotted circles. Cells are clearly held against a 20 nL/min flow rate with a calculated DEP.y of 0.2pN at 5Vpp. Video corresponding to this HEK-293 immobilization and accumulation is provided in Supplementary Video S3.

Separation of dead and live cells at the chosen frequency is shown in the time-lapsed images of Supplementary Figure S4. Cells were suspended in a 7% sucrose solution with osmolarity of 3.4 *μ*S/cm with 30 nM propidium iodide used as a stain for dead cells. Live cells remain in the nDEP trap with low field strength region while dead cells forced to the pDEP trap or region of high field strength between the half-ring and ground lines.

At the point of trapping, experimentally determined to be 60 *μ*m from the half-ring center, as indicated in Figure 5c, cells experience a Δ*ψ*_*mem*_ of ≈0.93 V for the 4V_PP_ trapping potential used, calculated from Equation 3, assuming no resistive drops along the electrode traces, sufficiently exceeding the electroporation threshold.

An important note here is that due to the size of the electrodes, multiple cells can be immobilized within a single trap. In this case, cells held at specific locations within the trap will see a drop in the expected electric field due to shielding by adjacent cells. A cell located at the trapping point is negligibly affected by the presence of adjacent cells because at the point of trapping the electric field exists primarily in the z-dimension, as shown in Figure 3. This is true of all cells, which trap concentrically to the ring electrode. However, cells which trap upstream of the gradient minimum, will see a notable drop in electric field intensity. In implementation, solution cell concentration should be kept low to ensure no particles trap within this region. Note that with scaling in the single-cell version of this trap, these issues will no longer be of concern.

### AC electroporation of HEK-293 cells

**Figure 6:**
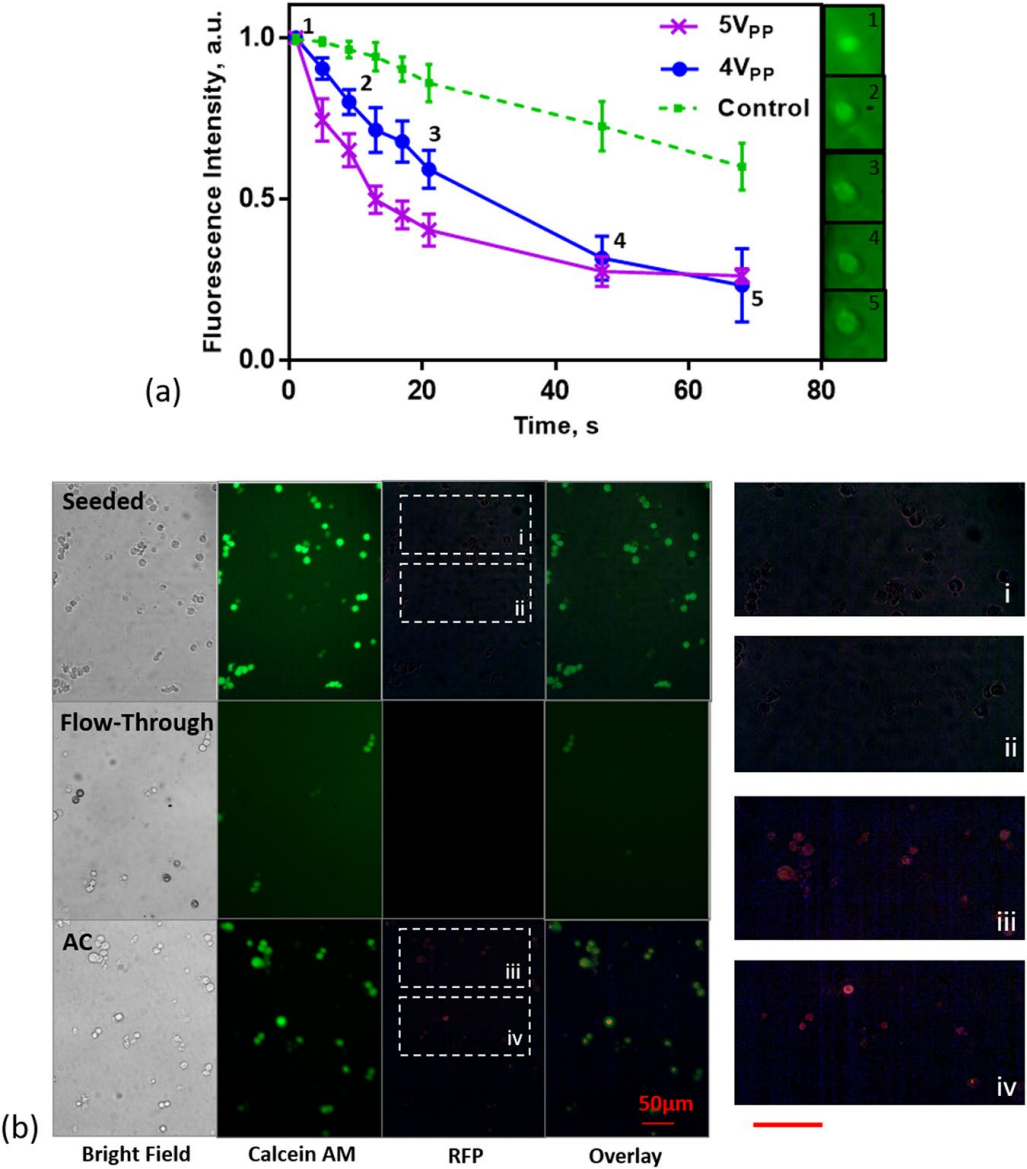
(**a**) Decrease in Calcein AM fluorescence intensity with time in nDEP trap indicating AC electroporation. Full size images corresponding to cropped subset are provided in Supplementary Figure S5. (**b**) Brightfield, Calcein AM and RFP channels for cultured cells after 24 hours for the control and electroporation conditions. Zoomed in images of RFP channels are provided in i,ii,iii and iv for clarity. Scale bar: ≈50 *μ*m.

As a marker of poration, we employ previously published methods^21,44^ examining the rate of Calcein AM leaching from the cells at two different AC amplitudes, 4- and 5- V_PP_. Calcein AM is a cell permeant dye which interacts with the esterases produced during cell metabolism. Under each condition, 5 cells were examined for the rate of Calcein AM leaching while subject to a DEP force in the trapping region, defined as the area within the ring trap where the cell is subject to an *E*_*RMS*_ greater than 1 kV/m and 1.3 kV/m for the two signals respectively. Figure 6a plots the fluorescence intensity of the cells normalized to the background intensity against time confirming electroporation at the chosen 4V_PP_ amplitude with simultaneous nDEP trapping of cells. The process is repeated for a 5V_PP_ signal which shows an increased rate of leaching confirming AC-field induced poration in the device; the measured photobleaching rate of the dye (control) is also provided for reference confirming fluorescence loss is indeed due to poration. Supplementary Video S4 provides visual confirmation of Calcein AM leaching from the cells.

For demonstration of simultaneous trapping and AC electroporation for delivery of exogenous material, we transiently transfect HEK-293 with a plamsid containing a red fluorescent protein tag which will cause transfected cells to exhibit red fluorescence using the procedure outlined in the Methods section below. For the seed only condition, cells were seeded directly into the 96-well plate with plasmid at 3.4 *μ*g/*μ*L. For the flow-through condition, cells were flowed through the device at 20 nL/min at 2 minutes under no applied field and subsequently collected at 5 *μ*L/min for 1 minute. Figure 6b shows the bright field, Calcein AM and RFP channels and their overlay for the controls and AC electroporation conditions. For clarity, zoomed in images of the RFP channel are provided in i,ii,iii and iv, corresponding to the labeled regions of Figure 6b. The electroporation condition shows transfection of ≈80% of the viable cells in the imaged area with control conditions showing no transfection after 24 hours.

### Scaling for single cells

**Figure 7:**
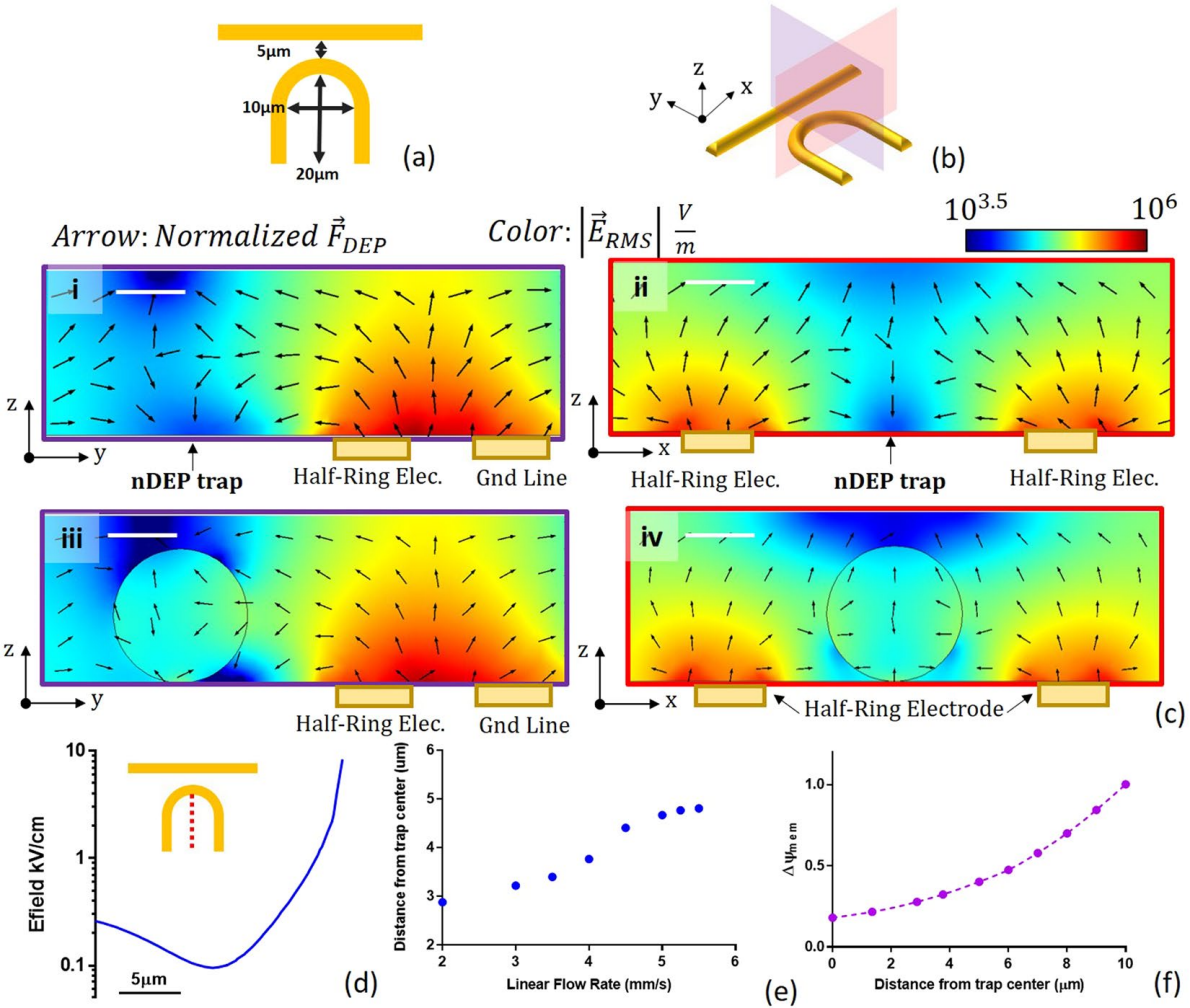
(**a**) Scaled trap geometry for single-cells. (**b**) Trap cross sections shown in (**c**) with y-z planes indicted in red and x-z planes indicated in purple. (**c**) Trap cross sections indicating location of nDEP trap along the y (c-i) and x (c-ii) dimensions and distortion of electric field in the same planes caused by presence of a cell (c-iii,iv) for an 8V_PP_ applied voltage. (**d**) Electric field along the half-ring central line indicating location of minimum field. (**e**) Simulated trapping point from half-ring center for various flow rates and (**f**) theoretical change in transmembrane potential in trap area for 7 kHz applied signal. Scale bar: 5 *μ*m.

The results presented above were obtained using larger half-ring electrodes which enable simultaneous trapping and electroporation of multiple cells, however there is a strong scaling argument that justifies this design for single-cell trapping and AC electroporation. We illustrate this through numerical simulation of a scaled version of this trap with dimensions shown in Figure 7a, which is 10x smaller. The planes indicated in Figure 7b and shown in Figure 7c(i–ii) indicate the location of the nDEP trap approximately 8 *μm* from the trap edge for a 8V_PP_, 7 kHz applied signal. The DEP force on the cell at this point is simulated to be 8.9pN which is sufficient for trapping with linear flow rates below ≈5.5 mm/s. The trap self-limits capture of single cells due to the distortion in the electric field caused by the presence of a cell, as indicated in Figure 7c(iii–iv). Here the electric field gradient resulting in an nDEP trap is no longer present with the minimum now located upstream of the cell. Within the trap area, the change in Δ*ψ*_*mem*_ will meet the ≈0.4 V electroporation threshold for flow rates greater than 5 mm/s (Fig. 7e,f).

## Conclusion

We present a simply fabricated flow-through device for simultaneous nDEP trapping and AC electroporation. We demonstrate the device for simultaneous trapping and transfection of HEK-293 cells with a plasmid for FusionRed RFP expression. The combination of the two phenomena has two primary advantages over a platform utilizing separate excitations. First holding of the cell while material flows increases the chance that exogenous material come in proximity to and enter cells. Second, physical confinement of the cells to the coplanar of the electrodes is not necessary, ensuring cells experience the intended electric field. Furthermore, we propose that this method provides better control over unwanted lysis as cells are pushed to regions of low electric field. The next steps involve comprehensive electroporation studies using this platform on different cell types and transfection with different material. Holding time within the trap will be optimized for maximum transfection efficiency. This will be the basis of a future publication.

Here we presented results for a version of the trap capable of isolating and transfecting multiple cells; these dimensions were chosen for ease of fabrication in the available facilities. However there is a strong single-cell scaling argument for the designed electrode structure which we illustrate through numerical simulation. Currently the flow rate in the system is limited to a few nL/min and is due to fabrication limitations which limits spacing between electrodes and consequently the electric field gradient which can be created. In a single-cell version of this trap, larger nDEP forces will be exerted on cells allowing for higher flow rates. This paper is an important first step towards realizing a complete platform for contactless single-cell separation from a conglomerate with subsequent transfection and lysis capability through adjustment of the applied electric field. Such a platform has numerous applications in targeted drug development, cancer genomics and pathway discovery.

## Methods

### Fabrication

The platform is fabricated using standard photo-lithographic lift-off techniques. Briefly, SPR-220-3 photoresist is spin coated onto a standard 100 mm SiO_2_ wafer and is exposed to create the electrode pattern. Titanium (Ti) and gold (Au) are subsequently sputtered to deposit the metal electrodes using 10 nm Ti as an adhesion layer, followed by a 20 nm co-sputter and a final 150 nm Au sputter to form the final Au electrodes. The SPR-220-3 resist is lifted off in acetone under sonication leaving the electrode pattern. A diamond scribe is used to separate devices. A PDMS channel is subsequently formed using a laser cut polyurethane sheet with a 70 *μ*m thickness as a mold and is bonded to the electrodes using ambient air plasma.

A porous PEG thin film is subsequently formed on all surfaces to prevent cell adhesion using a PEG 80 solution which is pumped into the PDMS channel and left overnight. Excess PEG is flushed out of the device using DI water.

### Finite element analysis of particle trajectories

Finite element analysis was completed with COMSOL Multiphysics simultaneously using the electrostatics, creeping flow and particle tracing modules. A Dirichlet boundary condition with applied voltage was imposed at both electrodes. A no-slip condition was imposed at the electrode-glass interface and top channel surface which assumes simple Poiseuille flow. Infinite inlets and outlets were assumed at the two ends of the channel. Particles were introduced as constant time intervals. Here the influence of the electric double layer (EDL) on simulated electric fields was neglected as the associated capacitance will reduce the simulated field by less than 7%. Supplementary Section S5 describes in detail the determination of this capacitance value using electrochemical impedance spectroscopy (EIS).

The equivalent complex permittivities were calculated as outlined in Pethig (2017^)19^, for the HEK-293 cells using a double-shell model. Each cell component is assumed to contribute linearly to the effective permittivity and conductivity of the cell. A detailed explanation of the equations used for effective permittivity and CM factor determination is provided in the electronic supplementary information.

For simulation of the Clausius-Mosotti factor for dead cells, the assumption was made that plasma membrane conductance increased as the membrane degraded and was taken to be 100 S/m^19^. Because the formed PEG layer is porous and will exchange liquid with the surrounding medium, its dielectric properties were assumed to be equivalent to the 7% sucrose solution used in the described experiments.

### Microfluidic setup

A Fluigent pressure pump system (Fluigent, FlowEZ) is employed to maintain flow rate within the channel. The system regulates air pressure into a gas-impermeant fixed volume chamber containing the material to be flowed into the channel. A flow meter is placed in line with the fixed volume chamber and pressure pumps and is used as feedback by the pumps to regulate air pressure into the fixed volume chamber. Polyether ether ketone (PEEK) tubing with 200 um inner diameter and 700 *μ*m outer diameter are used for fluid flow in the setup. 15 mL conical centrifuge tubes with a Fluiwell 1C-15 cap are used as the fixed volume reservoir. An optical image showing the full test setup is provided in Supplementary Figure S6.

### Polystyrene particle force experiments

A Tektronix AFG3251C arbitrary waveform generator is used to generate the simultaneous DEP/AC electroporation fields. The location and strength of the nDEP trap is verified experimentally using 9.9 *μ*m polystyrene particles (ThermoFisher). Particles were suspended in a 7% sucrose solution (*σ* = 7.4 *μ*S/cm) and introduced into the device at a constant flow rate under a 20V_PP_, 1 MHz applied signal. Once a particle was held, flow rate was steadily increased in 4 s intervals until the particle was no longer held against the flow rate. At this point, the hydrodynamic drag force was assumed to be equal to the DEP force acting on the particle. Using the equation for hydrodynamic drag force on a particle under shear given in Equation 5 ^17^ the resulting DEP force is estimated. This equation is a modified form of Stoke’s law which accounts for particle-wall interaction. This method is preferred for estimation of the DEP force as the force will be altered by the presence of the particle close to the channel wall^45^. Here *η*_*m*_ is the viscosity of the medium, S is the shear rate and K is a coefficient accounting for the particle distance from the wall, taken to be 1.7005^17^. S is calculated using Equation 6 after determining the equivalent linear flow rate, v, rate based on the channel cross section^46^. Here, *d*_*h*_ is the hydraulic diameter used to estimate the effective diameter of the rectangular cross section given by Equation 7, where w and *h*_*c*_ are the width and height of the channel, equal to 1 mm and 70 *μ*m respectively^47^.

5 $${F}_{D}=-\,6\pi {R}_{c}{\eta }_{m}hSK$$

6 $$S=\frac{8v}{{d}_{h}}$$

7 $${d}_{h}=\frac{2w{h}_{c}}{w+{h}_{c}}$$

### Cell culture and plasmid transfection

For plasmid transfection, cells grown in Dulbecco’s Modified Eagle’s Medium (DMEM) with 10% fetal bovine serum and penicillin/streptomycin were trypsinized and suspended in 7% sucrose solution at a density of 3 × 10^6^ cells/mL. 13.47 *μ*g of plasmid vector tagged with RFP and 10 *μ*L of 4 mM Calcein AM was added to the cell solution. The mixture was introduced into the system under a 20 nL/min constant flow rate and plasmid transfection was carried out in the following sequence; (1) DEP trapping field of 4V_PP_ was left on for 2 minutes to simultaneously immobilize and electroporate cells at trap locations. Visual confirmation was obtained via concurrent imaging with a Leica DMIRB epifluorescent microscope. (2) Flow rate was increased to 5 *μ*L/min and cells were collected into 200 *μ*L of DMEM with 10% fetal bovine serum and 20 mM HEPES with penicillin/streptomycin in a 1.5 mL microcentrifuge tube. The entire contents containing ≈10,000 cells was transferred to a 96-well plate for culture in a 37 ºC incubator. The process was repeated 3x for each of the test and control conditions. Cells were cultured overnight and imaging of a 440 *μ*m × 330 *μ*m region of the cultured well was completed to determine if transfection had occurred. A detailed description of the plasmid design is provided Supplementary Section S7.

### Image acquisition and processing

Images are acquired using a Leica DMIRB epifluorescent microscope fitted with an AmScope MU300 CMOS microscope camera for image acquisition. A ≈577 nm ± 20 nm excitation filter is used for RFP imaging and a ≈470 nm ± 40 nm excitation filter is used for Calcein AM imaging.

All images were processed in ImageJ through separation of the red, green and blue channels with intensities normalized to background. The red channel is used for determination of FusionRed RFP intensity and the green channel is used for Calcein AM intensity. Cells locations were marked manually using the bright field image and viable cells were counted as those exhibiting Calcein AM fluorescence stronger than 5%. Transfected cells were marked as those exhibiting both RFP and Calcein AM intensity greater than 5%.

## Supplementary information

Supplementary Information

Supplementary Video 1

Supplementary Video 2

Supplementary Video 3

Supplementary Video 4
