# Supplementary material for: A flow through device for simultaneous dielectrophoretic cell trapping and AC electroporation
Source: Sci Rep. 2019 Aug 19;9:11988. doi: 10.1038/s41598-019-48198-x (PMC6700080; doi:10.1038/s41598-019-48198-x)
Supplement: Supplementary file 1 — Supplementary Information [file 41598_2019_48198_MOESM1_ESM.pdf]

Supplementary Information for *A flow through device for simultaneous dielectrophoretic cell trapping and AC electroporation*

Meera Punjiya, Hojatollah Rezaei Nejad, Juanita Mathews, Michael Levin and Sameer Sonkusale

S1: Parameters and Models for FEA Simulation

S1.1: Polystyrene Particles

The real portion of the CM factor for the polystyrene beads in the 7% sucrose solution is shown in Figure S2, indicating that the particles will experience nDEP at 1MHz. The polystyrene beads were assumed to be homogenous with particle and medium conductivities given by the parameters in Table S1.

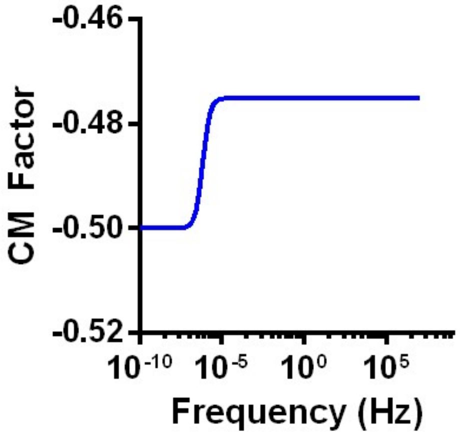

Figure S1: CM factor for polystyrene particles in 7% sucrose solution.

Description	Parameter	Value
Particle Conductivity (S/m)	σ_p	6.7e-14
Particle Permitivity	ϵ_p	2.7
Medium Conductivity (S/m)	σ_m	3.4e-4
Medium Permitivity	ϵ_m	80
Particle Radius (m)	R_p	4.95e-6

Table S1: Parameters used for 9.9μm polystyrene particle simulation.

S1.2: CM Factor Determination for HEK-293 Cells

A double-shell model of Figure S2 was used for the HEK-293 cells to capture the behavior of both live and dead cells. Here we assume that the cell nucleoplasm, nuclear membrane, cytoplasm and plasma membrane contribute equally to the effective cell permittivity. The effective complex cell permittivity is given by equation 3 where the effective permittivities for the nucleus, ϵ_{neff}, and the nucleus and cytoplasm composite, ϵ_{nc}, are given by equations 1 and 2 respectively [1]. The parameters R_c, C_{mem}, G_{xpm}, ϵ_{cyt}, σ_{cyt}, σ_{ne}, ϵ_{ne}, d_{ne}, ϵ_{np}, σ_{np} are as indicated in 2 and represent the cell radius, plasma membrane capacitance and conductance, cytoplasm permittivity and conductivity, nuclear envelope conductivity and permitivity, the thickness of the nuclear envelope and the permitivity and conductivity of the nucleoplasm respectively.

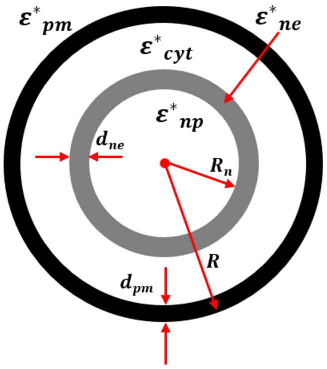

Figure S2: Double-shell model used for HEK 293 cells. Adapted from [1]

$$\epsilon^*_{neff} = \frac{(2\epsilon^*_{ne} + \epsilon^*_{np})(R_n + d_{ne})^3 + 2(\epsilon^*_{np} - \epsilon^*_{ne})R_n^3}{(2\epsilon^*_{ne} + \epsilon^*_{np})(R_n + d_{ne})^3 - (\epsilon^*_{np} - \epsilon^*_{ne})R_n^3}\epsilon^*_{ne} \tag{1}$$

$$\epsilon^*_{nc} = \frac{(2\epsilon^*_{cyt} + \epsilon^*_{neff})(R_c - d_{pm})^3 + 2(\epsilon^*_{neff} - \epsilon^*_{cyt})(R_n + d_{np})^3}{(2\epsilon^*_{cyt} + \epsilon^*_{neff})(R_c - d_{pm})^3 - (\epsilon^*_{neff} - \epsilon^*_{cyt})(R_n + d_{np})^3}\epsilon^*_{cyt} \tag{2}$$

$$\epsilon^*_{ceff} = \frac{(2\epsilon^*_{pm} + \epsilon^*_{nc})R_c^3 + 2(\epsilon^*_{nc} - \epsilon^*_{pm})(R_c - d_{pm})^3}{(2\epsilon^*_{pm} + \epsilon^*_{nc})R_c^3 - (\epsilon^*_{nc} - \epsilon^*_{pm})(R_c - d_{pm})^3}\epsilon^*_{pm} \tag{3}$$

$$\epsilon^*_x = \epsilon_x - i\frac{\sigma_x}{2\pi f} \tag{4}$$

The Clausius-Mosotti factor was subsequently determined using equation 5, where ϵ^*_m is the complex medium permittivity determined by the DC medium permittivity, ϵ^*_m, and the frequency dependence of the medium conductivity, σ^*_m. The values of all parameters used are as indicated in Table S2.

$$CM = \frac{\epsilon^*_{ceff} - \epsilon^*_m}{\epsilon^*_{ceff} + 2\epsilon^*_m} \tag{5}$$

Description	Parameter	Value	Reference
Cell Radius (m)	R_c	5e-6	[2]
Plasma Membrane Capacitance (μF/cm^2)	C_{mem}	1.1	[2]
Plasma Membrane Thickness (m)	d_{pm}	7e-9	[3]
Plasma Membrane Conductance - Live (S/m^2)	G_{lpm}	0.2	[1]
Plasma Membrane Conductance - Dead (S/m^2)	G_{dpm}	100	[1]
Cytoplasm Permitivity	ϵ_{cyt}	60	[3]
Cytoplasm Conductivity (S/m)	σ_{cyt}	0.5	[3]
Nuclear Envelope Conductivity (S/m)	σ_{ne}	1e-4	[1]
Nuclear Envelope Permitivity	ϵ_{ne}	28	[1]
Nuclear Envelope Thickness (m)	d_{ne}	40e-9	[1]
Nucleoplasm Permitivity	ϵ_{np}	52	[1]
Nucleoplasm Conductivity (S/m)	σ_{np}	1.35	[1]
Medium Permitivity	ϵ_m	80	
Medium Conductivity (S/m)	σ_m	.00034	*measured*
Medium Viscosity (Pa-s)	η_m	1.07e-3	[4]

Table S2: Parameters used for double-shell modeling of HEK-293 cells for determination of CM factor.

S2: HEK-293 Cells Under pDEP

For coarse verification of the chosen double-shell model, cells were subject to a analystically determined pDEP to ensure cells migrated from the inner half-ring to the high field region between the half-ring and ground line. Cells under pDEP are shown in Figure S3 with a 1MHz, 20Vpp force under constant flow.

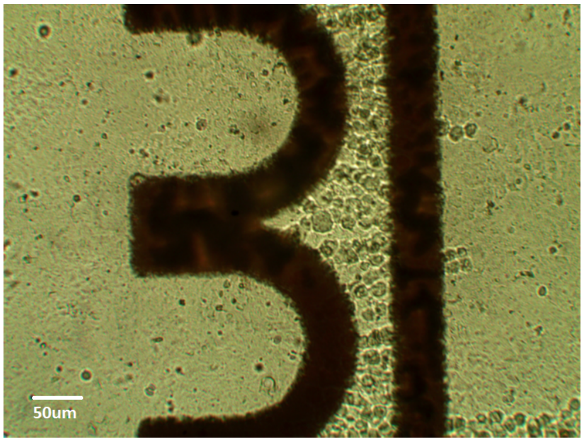

Figure S3: HEK-293 cells under pDEP force at 1MHz, 20Vpp applied voltage

S3: Separation of Live and Dead HEK-293 Cells

A frequency of 7kHz was chosen as there was a clear separation of dead vs. live cells in the chosen medium. The time-lapse image of Figure S4 indicates the separation of the live (clear) and dead (red) cells. Live cells experience nDEP while dead cells experience pDEP at the same frequency. Dead cells are stained using propidium iodide before introduction into the system.

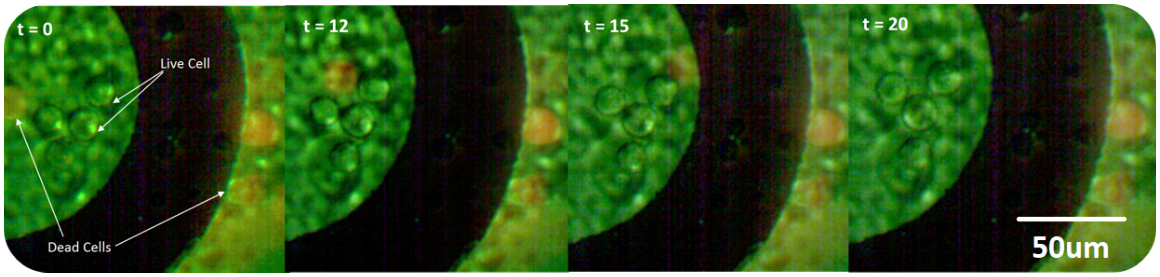

Figure S4: Separation of live vs. dead cells at 7KHz. Live cells experience nDEP staying on the inside of the half-ring trap while dead cells are pushed to high field strength between the right and ground line.

S4: Full Size Images of HEK-293 Cell Electroporation

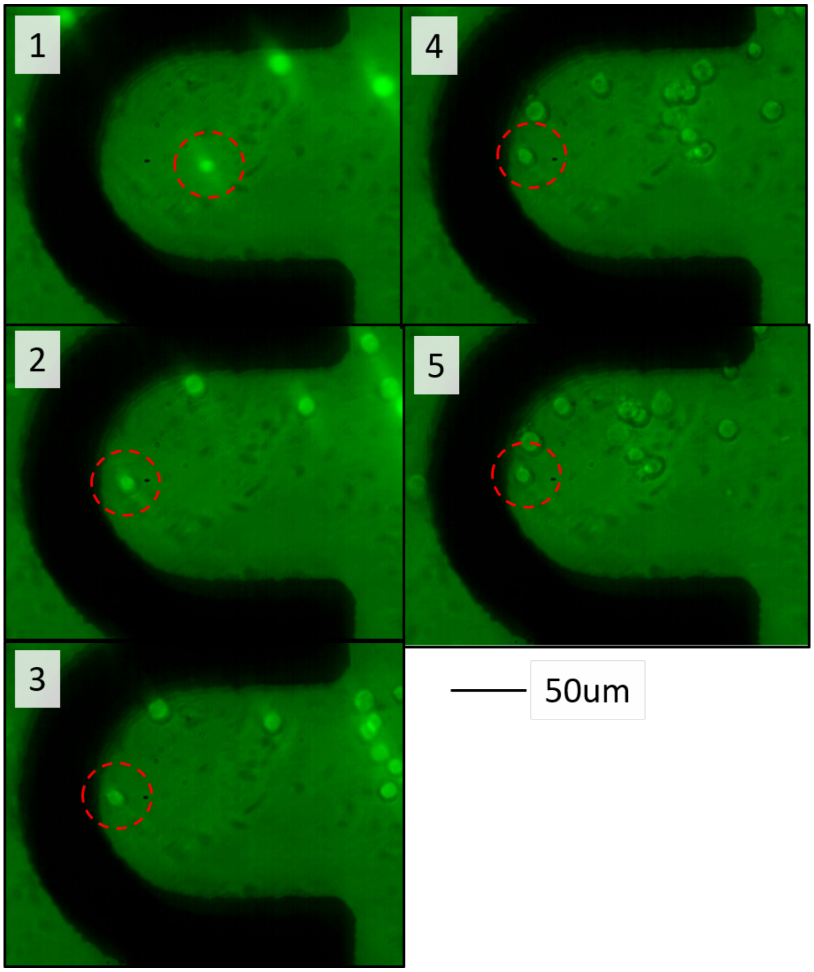

Figure S5: Time lapsed images showing leaching of calcein AM dye. The number in the full size images correspond to the cropped subset and times indicated in Figure 6a.

S5: Determination of the Double Layer Capacitance

The 7% sucrose solution used in the experiments here results in an electric double layer (EDL) that will negligibly affect the simulated electric field values. To demonstrate this, using electrochemical impedance spectroscopy (EIS) an approximate value for the EDL capacitance (C_{DL}) was extracted from a fit of the acquired Nyquist plot. This data was acquired using the two electrode sets as the counter and working electrodes with the sucrose solution used for electroporation experiments. Here the cumulative C_{DL}, modeled by a constant phase element (CPE)[5], is extracted to be 29nF. At 7kHz, this element has an impedance of approximately 6.9kΩ. The solution resistance here is 97kΩ extracted from the same model which results in an electric field attenuation of approximately 6.6% due to the C_{DL} present at both electrodes and was thus neglected from simulation.

The model, shown in Figure S6, is adapted from [6] and shows the double layer capacitance, C_{DL}, modeled as a constant phase element (CPE) which couples the electrode to the solution in series with a parallel resistor and capacitor which models the solution resistance (R_s) and capacitance (C_s). This model is valid as there is no charge transfer between the bare gold electrodes and the solution. The fitted model is shown in green against the acquired data for clarity.

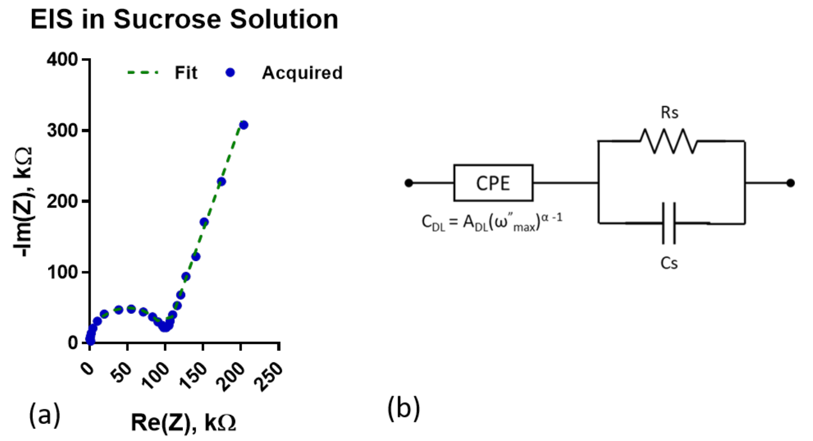

Figure S6: (a) Nyquist plot of fabricated gold electrodes in 7% sucrose solution (V = 20mV). (b) Model used for determination of the double-layer capacitance, C_{DL}. R_s and C_s are the resistance solution and capacitance respectively.

S6: Test Setup

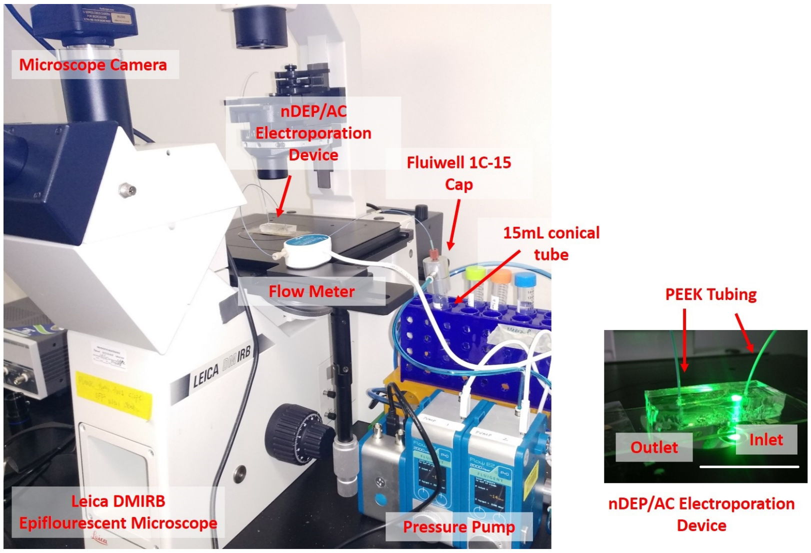

Figure S7: Microfluidic test setup of nDEP/AC electroporation device indicating imaging system, Fluigent pressure pumps and fluid reservoirs. Scale bar: ≈1.5 inches.

S7: Plasmid Design

The Fusion Red portion of FusionRed-pBAD [7] (Addgene plasmid # 54677) was infused (Clontech) into a pENTR1A vector (Invitrogen) containing a CAG promoter. The resulting pENTR1A vector was then LR cloned using the Gateway system into a hyperactive piggyBac transposase-based, helper-independent and self-inactivating delivery system, pmhyGENIE-3 containing a neomycin resistance gene for selection.

References

[1] Ronald R Pethig. *Dielectrophoresis: Theory, Methodology and Biological Applications*. John Wiley & Sons, 2017.

[2] Luc J Gentet, Greg J Stuart, and John D Clements. Direct measurement of specific membrane capacitance in neurons. *Biophysical journal*, 79(1):314–320, 2000.

[3] Rebecca Soffe, Sara Baratchi, Shi-Yang Tang, Mahyar Nasabi, Peter McIntyre, Arnan Mitchell, and Khashayar Khoshmanesh. Analysing calcium signalling of cells under high shear flows using discontinuous dielectrophoresis. *Scientific reports*, 5:11973, 2015.

[4] James F Swindells. *Viscosities of sucrose solutions at various temperatures: tables of recalculated values*, volume 440. For sale by the Supt. of Docs., USGPO, 1958.

[5] Piotr Zoltowski. On the electrical capacitance of interfaces exhibiting constant phase element behaviour. *Journal of Electroanalytical Chemistry*, 443(1):149–154, 1998.

[6] Shideh Kabiri Ameri, Pramod K Singh, Mehmet R Dokmeci, Ali Khademhosseini, Qiaobing Xu, and Sameer R Sonkusale. All electronic approach for high-throughput cell trapping and lysis with electrical impedance monitoring. *Biosensors and Bioelectronics*, 54:462–467, 2014.

[7] II Shemiakina, GV Ermakova, PJ Cranfill, MA Baird, RA Evans, EA Souslova, DB Staroverov, AY Gorokhovatsky, EV Putintseva, TV Gorodnicheva, et al. A monomeric red fluorescent protein with low cytotoxicity. *Nature communications*, 3:1204, 2012.
